# Fractal dimension as a measure of surface roughness of G protein-coupled receptors: implications for structure and function

**DOI:** 10.1007/s00894-012-1431-2

**Published:** 2012-05-29

**Authors:** Agnieszka A. Kaczor, Ramon Guixà-González, Pau Carrió, Cristian Obiol-Pardo, Manuel Pastor, Jana Selent

**Affiliations:** 1 Computer-Assisted Drug Design Lab, Research Programme on Biomedical Informatics (GRIB), PRBB, Dr Aiguader 88, Barcelona, 08003 Spain; 2 Department of Synthesis and Chemical Technology of Pharmaceutical Substances with Computer Modeling Lab, Faculty of Pharmacy with Division of Medical Analytics, Medical University of Lublin, 4A Chodźki St., Lublin, 20093 Poland; 3 Present Address: Intelligent Pharma, Barcelona Science Park, C/ Baldiri Reixac, 4, Barcelona, 08028 Spain

**Keywords:** Fractal geometry, G protein-coupled receptors, Ligand binding, Membrane cholesterol, Surface roughness

## Abstract

Protein surface roughness is a structural property associated with ligand-protein and protein-protein binding interfaces. In this work we apply for the first time the concept of surface roughness, expressed as the fractal dimension, to address structure and function of G protein-coupled receptors (GPCRs) which are an important group of drug targets. We calculate the exposure ratio and the fractal dimension for helix-forming residues of the β_2_ adrenergic receptor (β_2_AR), a model system in GPCR studies, in different conformational states: in complex with agonist, antagonist and partial inverse agonists. We show that both exposure ratio and roughness exhibit periodicity which results from the helical structure of GPCRs. The pattern of roughness and exposure ratio of a protein patch depends on its environment: the residues most exposed to membrane are in general most rough whereas parts of receptors mediating interhelical contacts in a monomer or protein complex are much smoother. We also find that intracellular ends (TM3, TM5, TM6 and TM7) which are relevant for G protein binding and thus receptor signaling, are exposed but smooth. Mapping the values of residual fractal dimension onto receptor 3D structures makes it possible to conclude that the binding sites of orthosteric ligands as well as of cholesterol are characterized with significantly higher roughness than the average for the whole protein. In summary, our study suggests that identification of specific patterns of roughness could be a novel approach to spot possible binding sites which could serve as original drug targets for GPCRs modulation.

**Figure:**
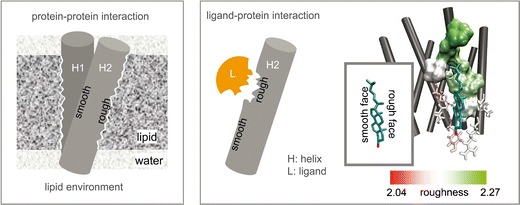
The significance of surface roughness for protein structure and function.

The significance of surface roughness for protein structure and function.

## Introduction

The concept of fractal geometry as a tool for describing objects in nature was elaborated by Mandelbrot [1]. A fractal can be considered as a rough geometric shape that can be divided into fragments, each of them representing a reduced copy of the whole. The extension of the concepts of fractal geometry toward life sciences has led to significant progress in understanding complex structural and functional features of tissues, cells and molecules. In particular, the concepts of fractal geometry have been applied to the description of structure and function of proteins [2]. The fractal geometry of the protein surface manifests itself in distinct global and local patterns of surface roughness. This peculiar feature of the protein surface determines the first level of communication with its surroundings in terms of diffusion, molecule recognition, and physical properties [3]. As a result, surface roughness can be a useful tool to describe and understand protein-protein and ligand-protein interactions [4–6].

**Fig. 1:**
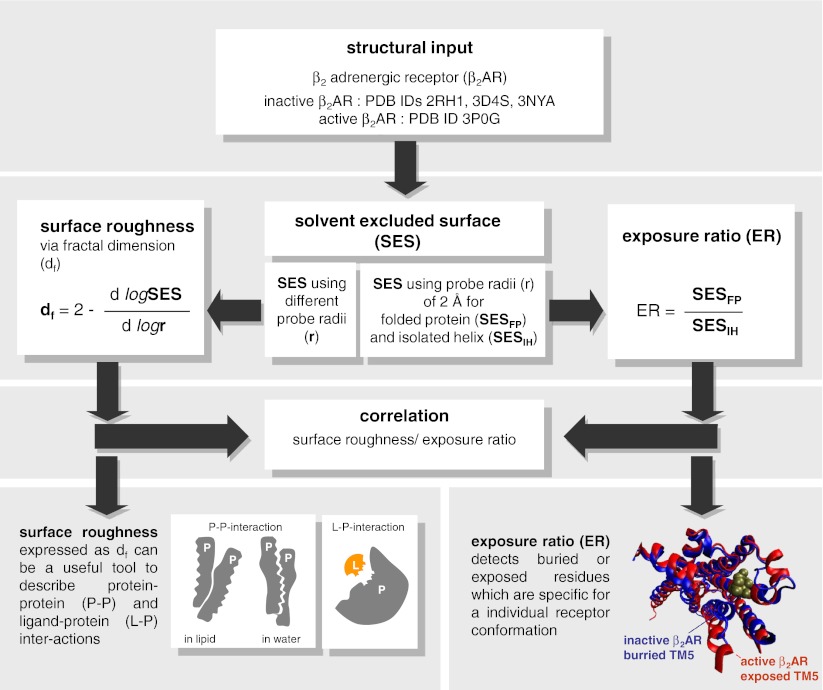
Workflow for the calculation of surface roughness and exposure ratio for different conformational states of the β_2_AR

Although the concepts of fractal geometry have been extensively used for the description of protein properties [2, 4–9], they have never been applied to G protein-coupled receptors (GPCRs). GPCRs are a group of medicinally important membrane proteins [10–13] which constitute drug targets for about 50 % of all lately launched drugs [14]. In the last years tremendous progress has been made in the research on GPCRs. X-ray structures of some GPCRs became available [15–21], including the structure of the β_2_ adrenergic receptor (β_2_AR) [20] and the adenosine A_2A_ receptor (A_2A_R) [21] in their active state. However, despite these efforts in understanding receptor structure and functioning (ligand binding, allosteric binding sites, protein-protein interaction), many aspects still remain unknown. Here, to address some of these aspects, we apply for the first time the concept of the fractal dimension for quantifying surface roughness of different conformational states of the β_2_AR in complex with an agonist, an antagonist and two partial inverse agonists (see workflow, Fig. 1). Quantitatively, surface roughness, expressed as the fractal dimension [7, 8], *d*_*f*_, is obtained by calculating solvent excluded surface (SES) using differently-sized probes (Fig. 1) [7]. We also use the SES to calculate the exposure ratio. Exposure ratio indicates how much a residue is exposed on the protein surface or buried in the protein interior. Our study shows that the pattern of SES and exposure ratio exhibits a periodic curve progression which is associated with the helical feature of the transmembrane (TM) domains in GPCRs. This finding is in agreement with previous observations for TM proteins [8]. Furthermore, we find that surface roughness as well as exposure ratio capture some characteristic features of active and inactive receptor conformations (see workflow, Fig. 1). Another important observation of our study is that surface roughness expressed as the fractal dimension is significantly different at the binding site of small molecules when compared to the average fractal dimension for the whole receptor. As a result, we suggest that the fractal dimension could be a useful parameter when searching for novel binding sites of GPCRs in drug discovery.

## Materials and methods

### Studied X-ray structures

Four X-ray structures of the β_2_AR in different conformational states were studied: in complex with partial inverse agonists carazolol (PDB ID: 2RH1 [15]) and timolol (PDB ID: 3D4S [16]), the neutral antagonist alprenolol (PDB ID: 3NYA [17]) and an agonist (PDB ID: 3P0G [20]).

For the purpose of further computations all accompanying molecules were removed. Hydrogen atoms were added and optimized with Yasara Structure [22, 23].

### Calculation of exposure ratio and the fractal dimension as a measure of surface roughness

Exposure ratio and fractal dimension were calculated following the methodology of Renthal [8].

#### Exposure ratio (ER)

First, the solvent-excluded surface was calculated for each atom from the atomic coordinates of the folded protein structure (SES_FP_, Fig. 1) using the MSMS software [24]. Then, analogous calculation was performed for isolated helices (SES_IH_, Fig. 1). In both cases a probe of 2.0 Å was used. The atomic contributions of SES were summed into values per residue. The exposure ratio of a given residue was obtained by the division of the corresponding SES value in the folded protein by its SES value in the isolated helix (SES_FP_/SES_IH_). The exposure ratio for a helix was calculated as the average of exposure ratios of the respective residues along with the standard deviation (SD). Exposure ratio was calculated for all helix-forming residues of the β_2_AR.

#### Surface roughness

Surface roughness is proportional to the fractal dimension, which can be calculated according to the following formula:

1 $$ {d_f} = 2 - \frac{{dlogSES}}{{dlogr}}, $$

where *r* denotes a probe size. Thus, in this case SES was calculated with a set of different probe radii: 1.2; 1.4; 1.6; 1.8; 2.0; 2.2; 2.4; 2.6; 2.8 Å. Following the methodology of Renthal [8], the value of a given residue was obtained by summing atomic contributions of residues forming a patch on the surface of the helix. This patch for a residue number N included sequence positions N-4, N-3, N, N + 3, and N + 4 which are neighboring residues resulting from the helical structure. The differential term in Eq. 1 was gained by plotting SES over probe size and application of linear regression to calculate the slope of the graph. The roughness of a helix was calculated as the average of roughness of respective residues, accompanied by the standard deviation (SD). Surface roughness was calculated for all the helix-forming residues of the β_2_AR except for the first four and the last four residues of each helix as described above.

In the case of exposure ratio and surface roughness data were processed using R [25] and Bio3d package [26].

### Mapping of roughness into receptors’ 3D structure

The obtained residual values of roughness were mapped into the protein 3D structure with VMD [27]. The residues forming small molecule binding sites were detected with the Molecular Operation Environment (MOE) [28] tool for identification of intermolecular contacts.

## Results and discussion

**Fig. 2:**
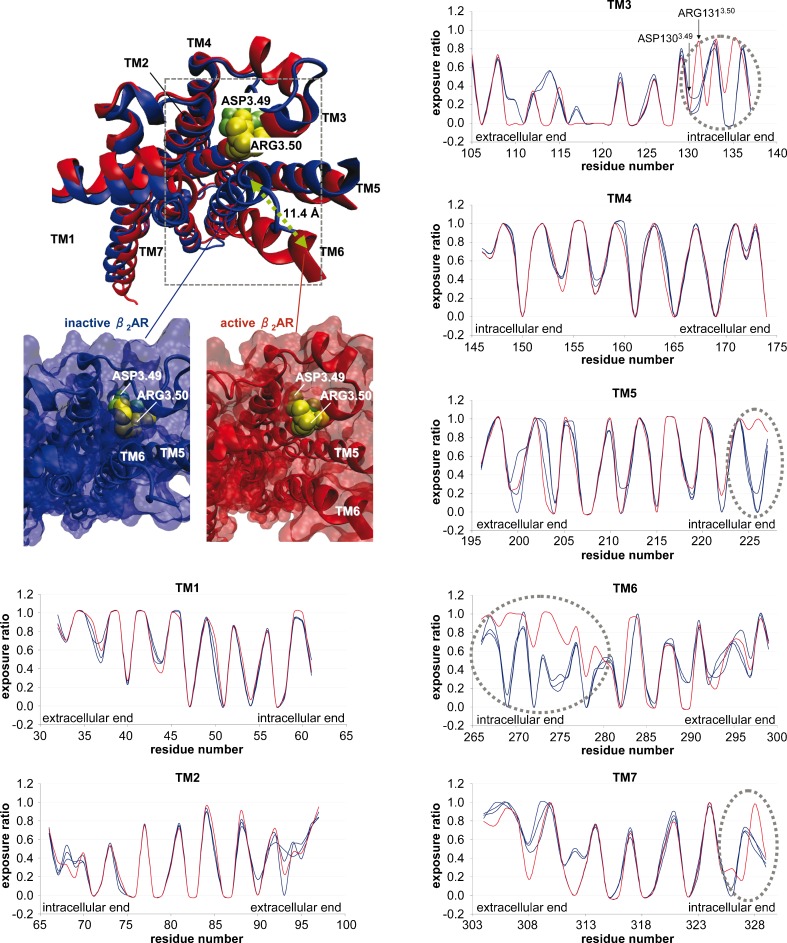
Top left: structural superimposition of the active (PDB ID: 3P0G) and the inactive (PDB ID: 2RH1) β_2_AR showing both receptors from the intracellular side as well as two structural insets in surface representation highlighting differences between the inactive and the active β_2_AR. Bottom left to right: exposure ratio of residues of TMs 1 – 7 for different conformational states of the β_2_AR. Blue lines correspond to inactive structures (PDB IDs: 2RH1, 3D4S, 3NYA) whereas the red line corresponds to the active structure (PDB ID: 3P0G)

In order to assess the concept of the fractal dimension as a tool for quantifying surface roughness (workflow, Fig. 1), we selected four different conformational states of the β_2_AR in complex with partial inverse agonists carazolol (PDB ID: 2RH1 [15]) and timolol (PDB ID: 3D4S [16]), the neutral antagonist alprenolol (PDB ID: 3NYA [17]) and an agonist (PDB ID: 3P0G [20]). The main structural difference between the different X-ray structures of the 7TM β_2_AR are found in the intracellular TM ends (Fig. 2, structural inset). In the active structure of the β_2_AR (PDB ID: 3P0G) the intracellular side of the receptor (site of G protein binding) is much more open in comparison to the inactive β_2_AR conformation (PDB IDs: 2RH1, 3D4S, and 3NYA, Fig. 2, structural inset). This is due to the 11.4 Å outward movement of TM6 present in the active structure. In the first step, we calculate surface roughness as well as exposure ratio for all four GPCR structures. In the second step, we compare active and inactive β_2_AR states to assign distinct roughness and exposure patterns to a particular receptor state. Finally, we investigate the characteristics of surface roughness at known small molecule binding sites of GPCRs.

### Exposure ratio of β_2_AR residues in different conformational states

The calculation of exposure ratio of helix-forming residues for each transmembrane segment is described in detail in the Materials and methods section. Table 1 presents the average exposure ratio of all seven TMs of four conformational states of β_2_AR.

**Table 1:** The average exposure ratio accompanied by the standard deviation (SD) of seven transmembrane segments of the β_2_AR in different conformational states

TM	3D4S (partial inverse agonist, timolol)	2RH1 (partial inverse agonist, carazolol)	3NYA (neutral antagonist, alprenolol)	3P0G (agonist)
TM1	0.6160 ± 0.3655	0.6666 ± 0.3538	0.6362 ± 0.3577	0.6550 ± 0.3419
TM2	0.3563 ± 0.3032	0.3629 ± 0.3012	0.3446 ± 0.3129	0.3612 ± 0.3209
TM3	0.3003 ± 0.2926	0.3127 ± 0.2967	0.2516 ± 0.3083	0.2921 ± 0.3290
TM4	0.6449 ± 0.3572	0.6312 ± 0.3559	0.6429 ± 0.3591	0.6119 ± 0.3589
TM5	0.5647 ± 0.3854	0.6047 ± 0.3693	0.5674 ± 0.3760	0.5960 ± 0.3813
TM6	0.4648 ± 0.2884	0.4878 ± 0.3017	0.4662 ± 0.3084	0.6238 ± 0.3395
TM7	0.5189 ± 0.3497	0.5092 ± 0.3401	0.4816 ± 0.3624	0.4579 ± 0.3577

*The SD is calculated over a helical structure which is characterized by a periodic exposure progression (alternation of buried and exposed residues) which consequently leads to relatively high SD values

The data show that independently from the conformational state of the β_2_AR, the most buried transmembrane segments are TM2 and TM3, whereas the most exposed ones are TM1, TM4 and to a lesser extent TM5, TM6 and TM7. Such a pattern of helix exposure is a characteristic signature of the GPCR architecture. The most buried segments, TM2 and TM3, are to the greatest extent involved in interhelical interactions within the 7TM bundle. The TMs which are more exposed may be involved in interactions with different molecules, including oligomerization with another GPCR. Indeed, the data about dimerization interfaces of GPCRs indicate most frequently the involvement of TM4 and TM5 [29]. TM5-TM6 interface occurs in the crystal structure of the recently obtained chemokine CXCR4 dimer, termed the first functional dimer of a GPCR [30]. For some receptors, TM1 [29] and TM6 [31] were also identified as the most probable oligomerization interface. Also, multiple oligomerization interfaces are possible as found in the semiempirical model of rhodopsin oligomer [32, 33]. This model involves TM1, TM2, TM4, and TM5 employing the most exposed TM regions for protein-protein contacts.

Interestingly, the highest difference in the exposure ratio for one particular TM among different conformational β_2_AR states is found for TM6 (Table 1). TM6 is much more exposed (exposure ratio of 0.6238) in the active state of the β_2_AR than in any of the inactive states (exposure ratio 0.4648 – 0.4878). This finding is connected with the 11.4 Å outward movement of the cytoplasmic end of TM6 (Fig. 2, structural inset) during the process of receptor activation [20]. Two other TMs that rearrange during the receptor activation are TM5 and TM7 [20]. Comparison of the exposure ratio of TM7 in the considered conformational states of the β_2_AR reveals that TM7 is more buried in the active conformation (exposure ratio of 0.4579) than in the inactive conformations (exposure ratio in the range of 0.4816 – 0.5189). In contrast, TM5 does not show a clear relationship at first glance: the exposure ratio of TM5 in the active state (0.5960) is comparable to the respective exposure in the β_2_AR-carazolol complex (0.6047) and slightly higher than of other inactive conformations (0.5647 and 0.5674). More details are obtained by plotting the exposure ratio of residues for each individual TM segment (TM 1 to 7) in different conformational of β_2_AR states (Fig. 2). First, a clear periodicity of exposure ratio is seen for all conformational β_2_AR states. This periodicity is a characteristic feature of the seven transmembrane α-helical bundle and results from the structural features of α-helices which possess periodicity of 3.6 residues per turn. It means that for transmembrane helices with one face buried in the protein core and the other exposed to the membrane, the residues at helix positions i, i + 3, i + 4 and i + 7 will lie on one face of the helix (e.g., buried in a protein interior) whereas i + 1, i + 2, i + 5 and i + 6 will lie on the other face (e.g., exposed to the membrane). As a consequence, a characteristic alternation of buried and exposed residues is observed when plotting the exposure of an α-helical bundle, as shown in Fig. 2.

Nevertheless, there are subtle differences in the exposure ratio of most residues when comparing an active conformation (PDB ID: 3P0G, Fig. 2, red line) with the inactive ones (PDB IDs: 3D4S, 2RH1, 3NYA, Fig. 2, blue lines), due to different receptor conformations. Major differences between active and inactive structures are found in intracellular ends of TM3, TM5, TM6 and TM7 whereas differences between the three inactive conformations (blue lines) are much smaller. For instance, we observe in TM3 that the exposure ratio of residue Arg131^3.50^ (superscripts indicate Ballesteros-Weinstein numbering for conserved GPCR residues) [34], which forms a part of the ionic lock at the intracellular TM end, is much higher (0.8817) in active than in inactive states (0.1464 – 0.2911). In contrast, the Asp130^3.49^ which is located next to Arg131^3.50^ and is also a part of the ionic lock has a similar exposure ratio in all four conformational states of β_2_AR. Rationalizing this data using the X-ray structures of the active and the inactive β_2_AR (Fig. 2, structural inset), we find that the 11.4 Å outward movement of TM6 is responsible for the observed exposure values of Arg131^3.50^ and Asp130^3.49^. A surface representation of the inactive β_2_AR (Fig. 2, structural inset down left) shows clearly that a 11.4 Å inward location of TM6 buries both residues Asp130^3.49^ (lime spheres) and Arg131^3.50^ (yellow spheres). In contrast, a 11.4 Å outward movement of TM6 as found in the active β_2_AR (Fig. 2, structural inset down right) results in an exposure of Arg131^3.50^ (yellow spheres) while Asp130^3.49^ (lime spheres) remains buried. The same TM6 outward movement contributes to the general high exposure ratio of the intracellular ends of TM3, TM5, TM6 and TM7 (highlighted by dashed circles, Fig. 2) of the active β_2_AR (red line) when compared to inactive β_2_ARs (blue lines). In this context, it can also be shown that different receptor types (β_2_AR, A_2A_R, and D_3_R) can be considered to be in a similar conformational state when complexed with an antagonist. This is due to these receptors exhibiting a similar residue exposure ratio (unpublished results). Therefore, besides an overall conserved tertiary structure, even side chain conformations of functionally important residues are evolutionary conserved.

Taking all data together, we suggest that functional residues of GPCRs adopt a different conformation in different conformational states of the same receptor, while they adopt a similar conformation in similar conformational states of different receptors.

### The fractal dimension (surface roughness) of the β_2_AR residues in different conformational states

Following the methodology of Renthal [8], we calculated the fractal dimension of helical residues of β_2_AR in different conformational states. Quantitatively, surface roughness, expressed as the fractal dimension, was obtained by calculating solvent excluded surface using nine differently-sized probes (see Materials and methods section). The regression coefficients (R^2^) of *d*_*f*_ versus the probe size were in all cases above 0.95, most often above 0.98. Table 2 shows the average roughness of seven TMs of the β_2_AR in the considered conformations.

**Table 2:** The average fractal dimension (surface roughness) accompanied by the standard deviation (SD) of seven transmembrane segments of the β_2_AR in different conformational states

TM	3D4S (partial inverse agonist, timolol)	2RH1 (partial inverse agonist, carazolol)	3NYA (neutral antagonist, alprenolol)	3P0G (agonist)
TM1	2.1244 ± 0.0303	2.1338 ± 0.0396	2.1139 ± 0.0271	2.1318 ± 0.0312
TM2	2.1009 ± 0.0257	2.1008 ± 0.0239	2.0964 ± 0.0329	2.1136 ± 0.0226
TM3	2.1322 ± 0.0439	2.1305 ± 0.0437	2.1324 ± 0.0486	2.1251 ± 0.0335
TM4	2.1168 ± 0.0345	2.1192 ± 0.0361	2.1317 ± 0.0429	2.1192 ± 0.0420
TM5	2.1214 ± 0.0469	2.1106 ± 0.0359	2.1245 ± 0.0472	2.1225 ± 0.0409
TM6	2.1134 ± 0.0341	2.1133 ± 0.0441	2.1111 ± 0.0392	2.1077 ± 0.0414
TM7	2.1335 ± 0.0321	2.1303 ± 0.0341	2.1306 ± 0.0423	2.1194 ± 0.0472

*The SD is calculated over a helical structure which is characterized by a periodic roughness progression (alternation of rougher and smoother residues) which consequently leads to relatively high SD values

**Fig. 3:**
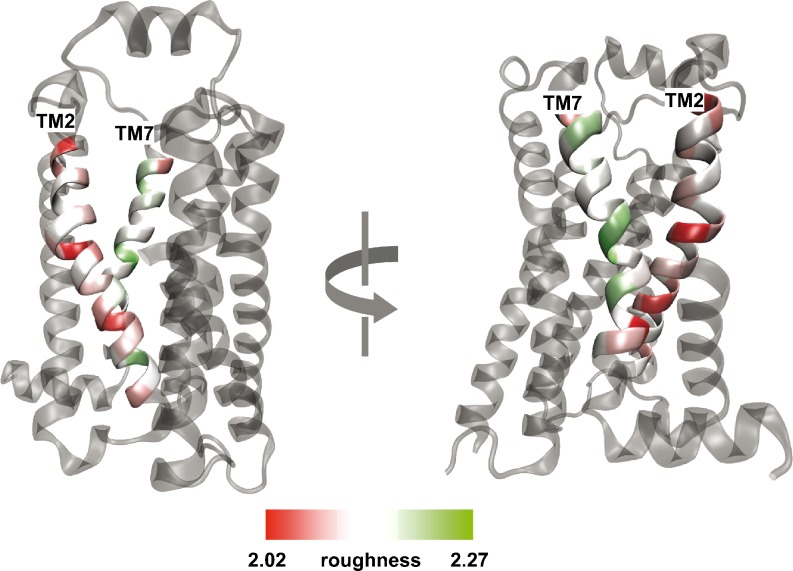
Roughness pattern of the alprenolol-bound β_2_AR (PDB ID: 3NYA): TM2 is smoother (reddish) than TM7 (greenish)

The roughness values (roughness range: 2.0964 – 2.1338, Table 2) reveal a tendency for certain TM helices to be smoother or rougher among different conformational states of the β_2_AR. For instance, it can be seen that TM2 is the smoothest whereas TM7 is the roughest one (Table 2). Interestingly, comparing the obtained surface roughness with the before calculated exposure ratio, we find that one of the smoothest helices, TM2 is simultaneously one of the most buried within our series of GPCRs (Table 1, Fig. 3). In contrast, the roughest TM7 is simultaneously the most exposed to the lipid environment (Table 1, Fig. 3). These findings are consistent with Renthal’s [8] reports that in the case of transmembrane α-helical proteins the smoothest residues are mainly buried and participate in intra-helical protein-protein interactions whereas the roughest residues are those that are exposed, interacting with membrane lipids. This can be justified, as follows, by thermodynamic reasons. All addressed crystal structures were obtained under comparable crystallization conditions, that is, in a lipid environment rich in fatty acids and cholesterol [15–21]. Such medium stabilizes the receptor in its naturally occurring 7TM architecture by mimicking the lipid membrane environment. Alkyl chains of lipids, when situated adjacent to a rough surface, adopt a thermodynamically favored conformation that is rich in kinks (i.e., gauche-trans-gauche). This is less common when alkyl chains are packed against a smooth surface instead [8]. Thus, the entropy of alkyl chains increases when they neighbor with a rough protein surface in comparison with a smooth one. Furthermore, by interacting along smooth surfaces, transmembrane helices exclude these regions from the interaction with lipids which decreases the free energy of a membrane. In contrast, smooth residues are likely responsible for interhelical contacts [8].

Besides TM2, TM3 is also a highly buried helix, but exhibits relatively high roughness (average fractal dimension in the range of 2.1251 – 2.1324). This is connected with the fact that TM3 contains various residues of the rough orthosteric binding site of the receptor (Trp109^3.28^, Asp113^3.32^, Val114^3.33^, Val117^3.36^, Thr118^3.37^), as discussed below.

**Fig. 4:**
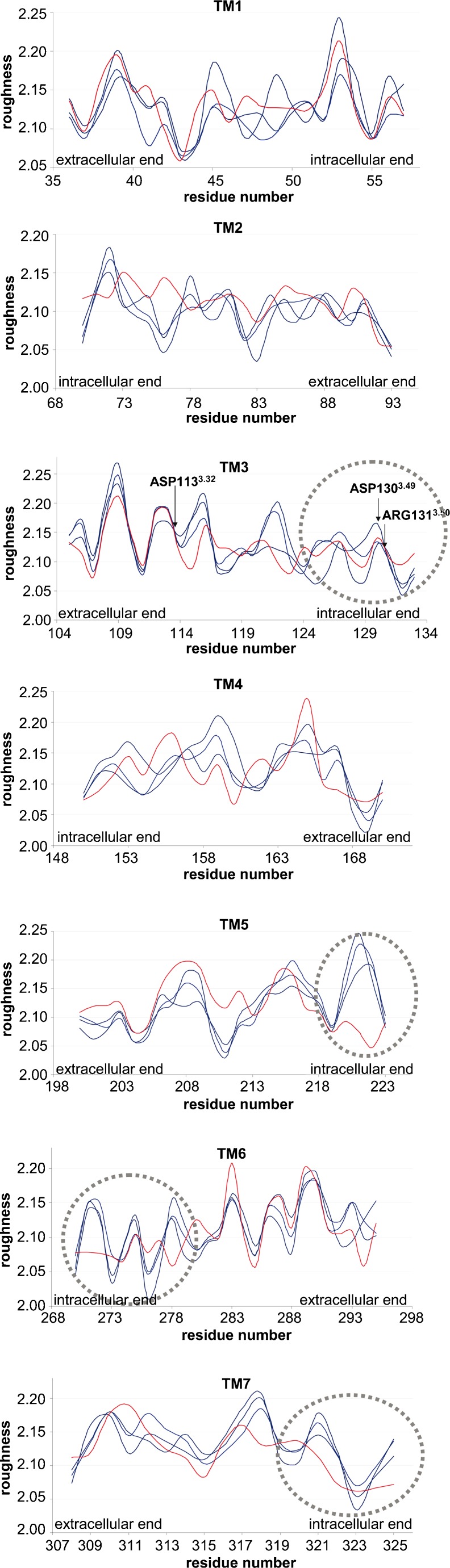
Fractal dimension (surface roughness) of residues of TMs 1 – 7 for different conformational states of the β_2_AR. Blue lines correspond to inactive structures (PDB IDs: 2RH1, 3D4S, 3NYA) and the red line – to the active structure (PDB ID: 3P0G)

The complete roughness pattern of each TM for β_2_ARs in different conformational states is plotted in Fig. 4. Similarly to the exposure ratio, roughness reveals a periodic pattern. This characteristic feature has to be ascribed to the peculiar structural properties of the 7TM helical bundle of GPCRs in which residues are alternating exposed to lipid (rougher) and neighbor helices (smoother) (see section: Roughness versus exposure ratio). Interestingly, the roughness profile between conformationally different structures (PDB IDs: 2RH1, 3D4S, 3NYA, 3P0G, Fig. 4) varies much more than the profile of the exposure ratio (Fig. 2). Even within the group of inactive structures (PDB IDs: 2RH1, 3D4S, 3NYA, Fig. 4, blue lines) the roughness profile is less conserved, particularly for TM 1 to 4. Apparently, the calculation of the surface roughness captures more detail of conformational differences even within inactive receptors than exposure ratio. Rather conserved roughness regions for the inactive structures (Fig. 4, blue lines) are found in TM5, TM6 and TM7. In this case, the active conformation of the β_2_AR (Fig. 4, red line) is clearly distinct from the inactive ones (blue lines). The roughness plot (Fig. 4) reveals flattened regions on the intracellular end of TM5, TM6 and TM7 for the active state of the β_2_AR (Fig. 4, red line) when compared to the inactive structures (Fig. 4, blue lines). Interestingly, the altered intracellular region consisting of the N-terminus of TM6 and C-terminus of TM5 and TM7 binds normally to the G-protein in the active β_2_AR (PDB ID: 3P0G). In contrast, in the inactive β_2_AR, the N-terminus of TM6 and the C-terminus of TM5 and TM7 is partially involved in intramolecular contacts as well as partially exposed to solvent interactions entering from the intracellular receptor side.

### Roughness versus exposure ratio

**Fig. 5:**
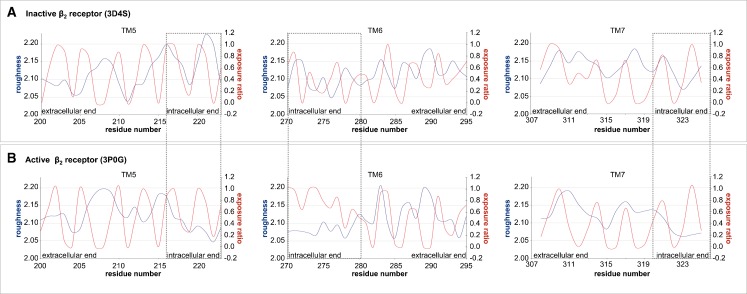
Roughness (blue) versus exposure ratio (red) of TM5, 6 and 7 of active and inactive forms of the β_2_AR

We have shown above for GPCRs that both the exposure ratio and the surface roughness expressed as the fractal dimension exhibit a periodic pattern (Figs. 2 and 4). Comparison of the pattern of the exposure ratio and the roughness for the inactive β_2_AR (Fig. 5) shows that in most cases the most exposed residues are simultaneously the most rough although a phase shift is observed [8]. However, meaningful exceptions occur in the active form of the β_2_AR (PDB ID: 3P0G), where residues of the intracellular ends of TM5, TM6 and TM7 are exposed but smooth (Fig. 5, labeled by boxes). These exceptions coincide with receptor regions which interact with the G protein-mimicking nanobody (PDB ID: 3P0G). Such smoothened and exposed protein surface is in agreement with Renthal´s findings who demonstrated that protein-protein interfaces are characterized with lower roughness than the average of transmembrane receptor [8].

Our results show that G-protein binding is responsible for the change in the roughness pattern on the intracellular end of TM helices. Moreover, our study suggests that roughness pattern is a valuable parameter to spot the interface of protein-protein binding as well as to allow distinguishing between active and inactive GPCRs.

The values of the exposure ratio and the fractal dimension presented in Tables 1 and 2 are characterized by high standard deviations. The standard deviation is a measure of the dispersion of a variable diversity. It shows how much the values of this variable vary from the average value. As both the exposure ratio and the fractal dimension exhibit periodic progression of high and low values, the values of these parameters are highly differentiated. In the case of the exposure ratio the values range from 0 (for completely buried residues) to 1 (for completely exposed residues) resulting in a high diversity within the data set and, in consequence, in high values of the standard deviation. Similarly, the diversity of the fractal dimension leads in turn to relatively high values of the standard deviation for this parameter too.

### Roughness of orthosteric and cholesterol binding sites of the β_2_AR

The surface roughness was calculated for classical orthosteric binding site and the experimentally observed cholesterol binding site at TM4. The cholesterol consensus motif at TM4 is present in about 26 % of GPCRs [16] and has been proposed to modulate protein function through direct and specific interaction with GPCRs [35].

**Fig. 6:**
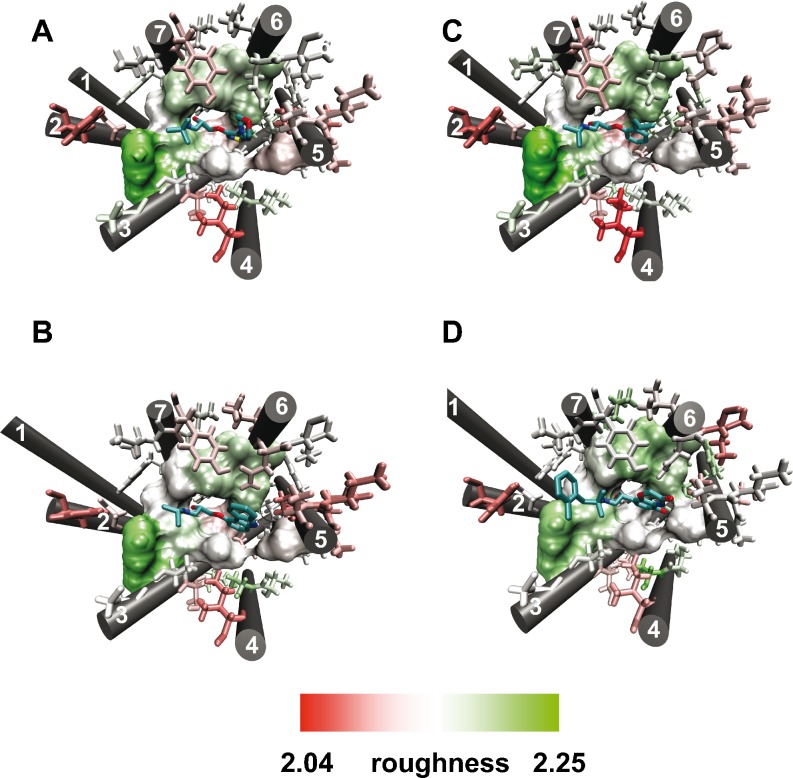
Roughness of the orthosteric binding site for different conformational states of the β_2_AR. **a** – **d**: orthosteric binding site in complex with inverse agonist timolol **a** and carazolol **b**, antagonist alprenolol **c** and agonist **d**. Residues in direct contact with the ligand are shown in surface whereas residues within 4.5 Å but not in direct contact are shown in licorice

**Fig. 7:**
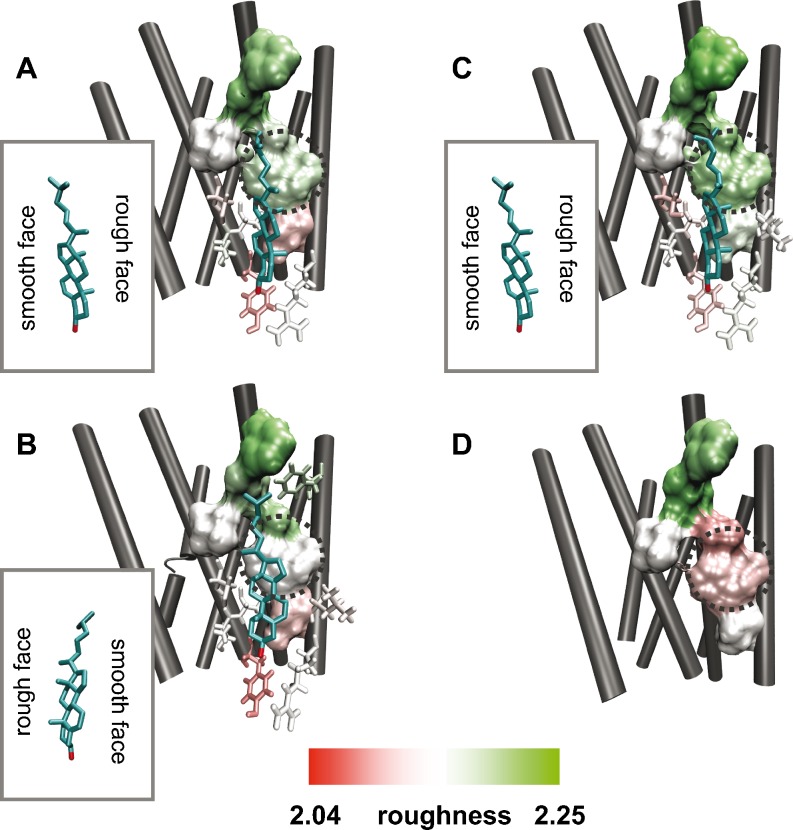
Roughness of the cholesterol binding site for different conformational states of the β_2_AR. **a** – **d**: orthosteric binding site in complex with inverse agonists timolol **a** and carazolol **b**, antagonist alprenolol **c** and agonist **d**. Residues in direct contact with the ligand are shown in surface whereas residues within 8 Å but not in direct contact are shown in licorice. The dotted circle highlights differences in the roughness pattern

Residues forming the orthosteric and the cholesterol binding sites were indentified using the MOE software [28] by taking into account a 4.5 or 8 Å-region around the ligand (Figs. 6 and 7, shown in licorice). Thereby, residues with direct ligand contacts were detected by applying the “protein contacts” tool of MOE software (Figs. 6 and 7, surface representation). Subsequently, roughness was calculated for the complete binding site omitting helix termini and loops; roughness values of direct-interacting residues are collected in Table 3.

**Table 3:** Average roughness of the β_2_AR in different conformational states accompanied by the standard deviation (SD) for the orthosteric and the cholesterol binding sites at TM4

	3D4S	2RH1	3NYA	3P0G
Complete receptor (exposed residues)	2.1215 ± 0.0347	2.1210 ± 0.0363	2.1227 ± 0.0416	2.1196 ± 0.0352
Orthosteric binding site Trp109^3.28^, Asp113^3.32^, Val114^3.33^, Val117^3.36^, Ser203^5.42^, Phe289^6.51^, Phe290^6.52^, Asn312^7.39^, Tyr316^7.43^	2.1567 ± 0.0501	2.1521 ± 0.0424	2.1572 ± 0.0577	2.1592 ± 0.0367
Cholesterol binding site (TM4) Val81^2.52^, Phe108^3.27^, Ile112^3.31^, Leu115^3.34^, Ile154^4.46^, Trp158^4.50^	2.1424 ± 0.0400	2.1430 ± 0.0411	2.1679 ± 0.0317	2.1303 ± 0.0394

The obtained data for orthosteric binding sites of different β_2_AR structures (Table 3) shows clearly that the average roughness values for residues interacting directly with timolol (PDB ID: 3D4S), carazolol (PDB ID: 2RH1), alprenolol (PDB ID: 3NYA), and an agonist (PDB ID: 3P0G) are higher (2.1521 – 2.1592) than the average for the whole receptor (exposed residues: 2.1196 – 2.1227). Furthermore, mapping the obtained roughness values onto the 3D structure using VMD [27] reveals that only direct-interacting residues (Fig. 6, a – d, surface representation) are rough (white to green). In contrast, not direct-interacting residues (Fig. 6, a – d, licorice representation), that are within 4.5 Å of the ligand, are mainly smooth (white to red color). Noteworthy, no significant roughness differences in the orthosteric sites (Fig. 6, a – d; Table 3: 2.1521 – 2.1592) are found between active (PDB ID: 3POG) and inactive (PDB IDs: 3D4S, 2RH1, 3NYA) conformations of β_2_ARs .

A similar tendency is found for the cholesterol binding sites at TM4 (Table 3). The roughness of this cholesterol binding site for the β_2_ARs adopts values from 2.1303 to 2.1679 which are also higher than the average of the receptor (exposed residues: 2.1196 – 2.1227). An interesting observation is that the active β_2_AR (PDB ID: 3P0G) has the lowest roughness of 2.1303 compared to the inactive β_2_ARs, from 2.1424 to 2.1679. Indeed, plotting the roughness onto all cholesterol binding sites reveals an altered roughness pattern for active (Fig. 7d) and inactive β_2_ARs (Fig. 7, a – c).

Thereby, the cholesterol binding site of the active β_2_ARs shows a smoother patch in the middle of the direct interacting residues (Fig. 7d, dotted circle) whereas the inactive ones are rougher (Fig. 7, a – c, dotted circle). This interesting finding can be linked to the fact that the active β_2_ARs has no cholesterol bound in the X-ray structure (PDB ID: 3P0G). The observed difference of roughness pattern points to a cholesterol binding-induced alteration of roughness. This is supported by another observation, that a different binding pose of cholesterol affects the roughness pattern. In the inactive β_2_ARs bound to timolol and alprenolol, the cholesterol interacts with a rougher protein surface through its “rough side”, that is, the side with methyl groups in the sterol ring (Fig. 7a and c). On the contrary, in the inactive carazolol-bound β_2_AR, the “smooth side” of the cholesterol interacts with a smoother protein surface (Fig. 7b). Therefore, the way cholesterol binds to the protein induces a particular roughness pattern. These differences indicate that surface roughness is a dynamic property of GPCRs.

## Conclusions

In this study, we apply for the first time the concept of surface roughness calculated as the fractal dimension for GPCRs. Surface roughness is an important property of proteins that can be linked to the architecture and function of proteins. Our study shows that both exposure ratio and fractal dimension are periodic features of GPCRs describing the 3D architecture of a 7TM helical bundle. Such periodicity is a result of the peculiar structural properties of the 7TM α-helical bundle of GPCRs, where residues are alternatively exposed to neighbor helices and lipid molecules. Thereby, buried residues interacting with neighbor helices are smooth whereas residues exposed to lipids are rather rough. Exceptions near the intracellular TM ends occur when the inactive β_2_AR adopts an active state. Hereby, roughness calculations clearly identify the region of G-protein binding, a key zone for GPCR signaling.

Moreover, our study indicates that also binding site for small molecules are detectable by roughness calculations. The classical orthosteric binding site as well as the experimentally observed cholesterol site at TM4 are significantly rougher than the average of the β_2_AR.

Finally, we prove that roughness is not a static but a dynamic property since the cholesterol binding/non-binding process, the cholesterol binding pose as well as G protein binding alter the surface roughness.

All in all, our results suggest that the calculation of roughness pattern may be a useful tool to identify protein binding sites as we determined that small molecule binding sites have higher fractal dimension than the average for the whole protein. Thus, our study suggests that studying surface roughness could become an interesting approach for detecting novel binding sites which is of high interest for drug discovery.

## Abbreviations

A_2A_R: Adenosine receptor type 2A
β_2_AR: β_2_ adrenergic receptor
CXCR4: CXC chemokine receptor type 4
*d*_*f*_: Fractal dimension
ER: Exposure ratio
GPCRs: G protein-coupled receptors
SD: Standard deviation
SES: Solvent excluded surface
SES_FP_: Solvent excluded surface of a folded protein
SES_IH_: Solvent excluded surface of an isolated helix
TM: Transmembrane
